# A case of eosinophilic dermatosis of hematologic malignancy with tissue-based Th2 cytokine profiling

**DOI:** 10.1016/j.jdcr.2022.09.020

**Published:** 2022-09-24

**Authors:** Suzanne Xu, Katelyn Singh, Jennifer McNiff, William Damsky, Jeffrey R. Gehlhausen

**Affiliations:** Department of Dermatology, Yale School of Medicine, New Haven, Connecticut

**Keywords:** Eosinophilia, Eosinophilic dermatosis, Interleukin 4, Interleukin 13, Lymphoma, Leukemia, T helper 2, DLCBL, diffuse large B-cell lymphoma, EDHM, eosinophilic dermatosis of hematologic malignancy, IL, interleukin

## Introduction

Eosinophilic dermatosis of hematologic malignancy (EDHM) is an uncommon cutaneous eruption occurring in the context of hematologic disorders. The disease presents as pruritic papules, plaques, blisters, or nodules that may resemble hypersensitivity to insect bites and demonstrates eosinophilic infiltration on histopathology.^1^ The pathogenesis of the condition is poorly understood but may result from an overproduction of Th2 cytokines stimulating eosinophil recruitment.^2^ We report a case of EDHM in a patient with a history of diffuse large B-cell lymphoma (DLBCL) featuring high levels of interleukin-13 (IL-13) in the skin, which has not previously been studied in this disease. Our findings suggest the role of excess IL-13 signaling in the development of EDHM.

## Clinical course

**Fig 1:**
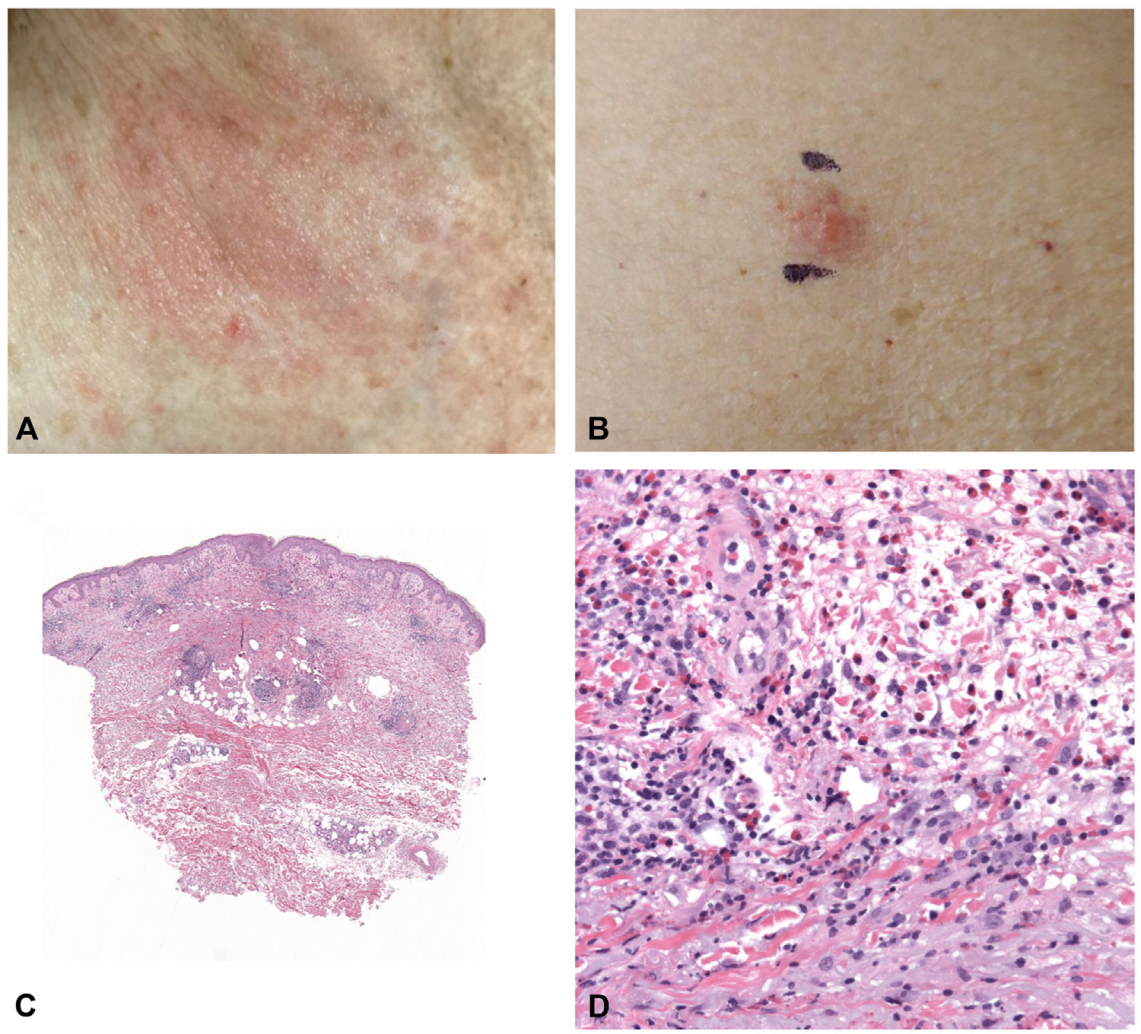
Clinicopathologic correlation of eosinophilic dermatosis of hematologic malignancy. **A,** Pink plaque on the right upper chest. **B,** Pink plaque on the leg (biopsied). **C,** Superficial and mid-dermal eosinophilic and lymphocytic infiltrate (40×, H&E). **D,** Numerous eosinophils (400×, H&E).

A 95-year-old woman with a past medical history of stage IV DLBCL in remission after treatment 6 months prior including R-CVP (rituximab, cyclophosphamide, vincristine, prednisone) and R-miniCHOP (rituximab, cyclophosphamide, doxorubicin, prednisone) presented with recurrent pruritic papules and plaques on the extremities and trunk for a 12-month duration (Fig 1, *A* and *B*). An identical rash had remitted with chemotherapy but recurred after completion. At the time of rash recurrence, she had an end-of-therapy positron emission tomography/computed tomography consistent with complete response to chemotherapy. She only took vitamin and iron supplements that she had been on for years and had not started any new medications preceding the rash recurrence. Punch biopsies of 2 plaques on the legs at the time of rash reoccurrence both showed a moderate, superficial to mid-dermal mixed inflammatory infiltrate with eosinophils (Fig 1, *C* and *D*).

**Fig 2:**
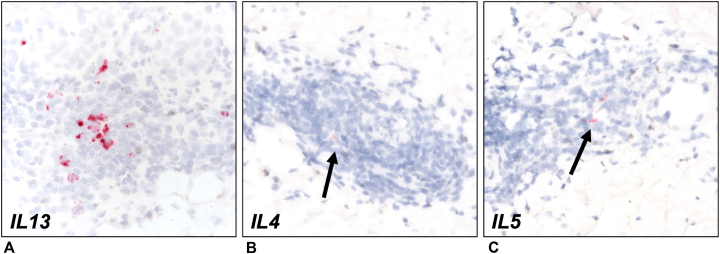
Cytokine RNA in situ hybridization showed significant *IL-13* staining (**A**), but faint *IL-4* (**B**) and *IL-5* (**C**) staining as indicated by *black arrows*. Images taken at 600× magnification.

She had no history of recent outdoor exposure or known arthropod bites. Laboratory testing with a complete blood count including absolute eosinophil count and lactate dehydrogenase were within normal limits. Bullous pemphigoid was ruled out by the negative direct immunofluorescence study on a perilesional biopsy. Given her past medical history, clinical findings, and histopathology, she was diagnosed with EDHM. Additional testing of the biopsies using in situ hybridization for *IL-4*, *IL-5*, and *IL-13* showed marked *IL-13* production in the areas of inflammation (Fig 2, *A*). There were only rare *IL-4–* and *IL-5*–positive cells (Fig 2, *B* and *C*). Positive controls were included for all assays. She was prescribed topical clobetasol 0.05% ointment, which led to resolution of lesions though did not prevent recurrences. She was offered narrow band ultraviolet phototherapy but declined treatment. She remained under active surveillance for DLBCL without any clinical concerns for recurrence, and laboratory findings including lactate dehydrogenase remained within normal limits.

## Discussion

EDHM is most commonly associated with chronic lymphocytic leukemia but may occur with a variety of other hematologic disorders including DLBCL.^3^ EDHM can be a challenging clinical diagnosis, as its presentation may mimic other conditions including arthropod bites and other inflammatory dermatoses.^4^ Patients may present with pruritic erythematous papules, plaques, and nodules as well as tense blisters.^1^ Biopsies typically show perivascular lymphocytic and eosinophilic infiltrate. The pathogenesis of EDHM is poorly understood, but hypothesized mechanisms generally focus on immune dysregulation associated with hematologic malignancy.^2^ IL-4, IL-5, and IL-13 are Th2 cytokines associated with the Th2-skewed inflammation (often with eosinophils) seen in disorders such as atopic dermatitis, allergy, and asthma.^5^ It has been proposed that an excess of IL-4 and IL-5 could lead to malignant B-cell proliferation and eosinophil proliferation and may play a role in the underlying neoplastic disease as well as the pathophysiology of EDHM.^4^ However, the levels of IL-13 have not been previously evaluated in EDHM. In our patient, in situ hybridization staining of the biopsies demonstrated elevated *IL-13* without significant expression of *IL-4* or *IL-5*, suggesting the possible importance of *IL-13* to the pathophysiology of EDHM.

Treatments of EDHM include topical steroids, systemic steroids, narrow band UVB phototherapy, oral antihistamines, dapsone, and antibiotics; only limited data exist regarding the efficacy of these different treatments.^1^^,^^4^ Studies of patients with EDHM have shown that patients respond to systemic steroids with or without concomitant topical steroids, but the majority relapse after steroids are discontinued.^1^ Dupilumab, a monoclonal antibody approved for atopic dermatitis that targets the alpha subunit of the IL-4 receptor to inhibit IL-4 and IL-13 signaling, has been used off-label in patients with EDHM with frequent flares and achieved sustained response for multiple months in select patients.^6^, ^7^, ^8^ Maglie et al^9^ selected a patient for dupilumab treatment based on the presence of inflammatory cells expressing IL-4 detected by immunohistochemistry in a lesional skin biopsy during a patient’s disease flare and observed complete lesion clearance after 6 weeks of treatment. Here, using RNA in situ hybridization, we demonstrate equivocal *IL-4* and *IL-5* staining but prominent *IL-13* staining which has not been previously demonstrated in EDHM (Fig 2).^10^ Recent therapeutic advances have led the development of novel IL-13–specific monoclonal antibodies, including lebrikizumab and the recently approved tralokinumab for atopic dermatitis. Our immunologic characterization of the patient’s lesions lends support to role of IL-13 in EDHM. Future studies are needed to evaluate if IL-13 is commonly elevated in the skin or serum of patients with EDHM and whether IL-13 targeted agents are efficacious in the treatment of steroid-resistant EDHM.

## Conflicts of interest

None disclosed.
